# Correlation between serum laminin levels and prognosis of acute myocardial infarction

**DOI:** 10.3389/fcvm.2022.936983

**Published:** 2022-07-22

**Authors:** Lou-Yuan Xu, Ling Xie, Jing Wang, Hai-Xiao Chen, Hong-Li Cai, Li-Jun Tian, Qing Zhang

**Affiliations:** ^1^Department of Cardiology, Second Affiliated Hospital of Nantong University, Nantong, China; ^2^Department of General Medicine, Second Affiliated Hospital of Nantong University, Nantong, China; ^3^Department of Critical Care Medicine, Nantong Third People's Hospital, Nantong University, Nantong, China

**Keywords:** acute myocardial infarction, cardiovascular disease, laminin, MACE, serum biomarker

## Abstract

**Background:**

Acute myocardial infarction (AMI) is a critical cardiovascular disease (CVD). Laminin (LN) is involved in the process of myocardial fibrosis and ventricular remodeling observed in AMI; however, there are currently no studies on the correlation between LN and AMI prognosis.

**Purpose:**

To explore the predictive value of serum LN levels for major adverse cardiovascular events (MACE) in patients, 6 months after an acute myocardial infarction.

**Methods:**

A total of 202 AMI patients who were hospitalized in the Department of Cardiology of the Second Affiliated Hospital of Nantong University between December 2019 and December 2020 were included. The observation endpoint was the occurrence of MACE. Univariate and multivariate logistic analyses were used to evaluate the relationships between the variables and endpoint. The predictive value of LN for MACE in AMI patients was assessed using receiver operating characteristic (ROC) analysis.

**Results:**

A total of 47 patients developed MACE. Univariate logistic analysis showed that smoking, emergency percutaneous coronary intervention (EPCI), age, cardiac troponin I (c-TNI) levels, N-terminal prohormone brain natriuretic peptide (NT-proBNP) levels, and LN levels were associated with the occurrence of MACE (*p* < 0.05). Multivariate logistic analysis showed that LN was an independent predictor of MACE (odds ratio [OR] = 1.021, 95%CI: 1.014–1.032, *p* < 0.001). According to the ROC curve, LN can be used as an effective predictor of MACE (AUC = 0.856, 95%CI: 0.794–0.918, *p* < 0.001). According to the cutoff value, LN>58.80 ng/ml (sensitivity = 83.00%, specificity = 76.80%) or LN>74.15 ng/ml (sensitivity = 76.6%, specificity = 83.2%) indicate a poor prognosis for AMI. Different cut-off values are selected according to the need for higher sensitivity or specificity in clinical applications.

**Conclusions:**

LN may be a predictor of MACE following AMI in patients and could be utilized as a novel substitute marker for the prevention and treatment of AMI.

## Introduction

Acute myocardial infarction (AMI) is one of the most common causes of death worldwide (1). Over the past few decades, improvements in cardiovascular treatments have significantly reduced the early death rate due to AMI. Despite the availability of new therapies, patients who survive an initial ischemic event are at a high risk of future adverse cardiovascular events (2). It is currently believed that ventricular remodeling is a key factor leading to poor prognosis in acute myocardial infarction (3), and the main pathological manifestation of myocardial remodeling is myocardial fibrosis (MF). Increased deposition of the myocardial extracellular matrix (ECM) and changes in collagen composition are closely related to myocardial fibrosis (4, 5). Endomyocardial biopsy is undoubtedly the gold standard for the diagnosis of myocardial fibrosis; however, its clinical application is limited. Therefore, biomarkers are considered an effective method for non-invasive detection of fibrosis. Several previous basic studies (6–8) have shown that laminin (LN) exists on the basement membrane of cardiomyocytes and fibroblasts (FBC), plays a role in connecting type IV, type I, and type III collagen, and participates in myocardial fibrosis. However, no study has reported a correlation between LN and the prognosis of patients with myocardial infarction. This study aimed to investigate the correlation between LN and the prognosis of patients with acute myocardial infarction and to explore the predictive value of LN to facilitate the early detection and intervention of disease progression in patients with myocardial infarction and improve the prognosis of patients.

## Methods

### Study population

We enrolled 202 patients (152 men and 50 women) with acute myocardial infarction who were admitted to the Department of Cardiology of the Second Affiliated Hospital of Nantong University from December 2019 to December 2020. All included cases were treated with standardized treatment. The patients were followed up for 6 months and then divided into an event group and a non-event group according to the occurrence of MACE. The study complied with the Declaration of Helsinki and was approved by the hospital ethics committee. Informed consent was obtained from all patients. The inclusion criteria were the diagnostic criteria for acute myocardial infarction: patients with troponin I levels above the 99th percentile of the upper limit of the reference value and at least one of the following: chest pain lasting >20 min or diagnostic serial ECG changes, including new pathological Q waves or ST segments and T wave changes (9). Exclusion criteria included fever, acute and chronic infection, malignant tumor, severe liver and kidney insufficiency, autoimmune disease, blood disease, stroke, myogenic disease, valvular heart disease, cardiomyopathy, heart failure, B-mode ultrasound-diagnosed internal carotid artery stenosis, renal artery stenosis, other major diseases, involuntary enrollment, mental and psychological diseases, and long-term bedridden patients who cannot take care of themselves.

Somking and EPCI (emergency percutaneous coronary intervention) will be included as factors in the analysis; According to the regulations of the World Healteh Organization, smoking is defined as those who have smoked continuously or accumulatively for 6 months or more since birth. EPCI refers to emergency percutaneous coronary intervention therapy, that is, coronary catheterization technique implemented within 24 h after the occurrence of acute myocardial infarction to dredge coronary stenosis or even occlusion of coronary vessel lumen, so as to improve myocardial blood perfusion.

### Detection of laminin

For patients diagnosed with acute myocardial infarction, 3 ml of venous blood was collected in the morning after 12 h of fasting. The serum was separated and stored at −20°C for future testing. The concentration of LN was detected by chemiluminescence immunosandwich method. One monoclonal antibody against LN was labeled ABEI and the other monoclonal antibody was labeled FITC. Samples, calibration, ABEI labeling monoclonal antibodies, FITC labeled monoclonal antibodies and Magnetic microspheres coated with goat anti-FITC antibody formed immunocomplex. Apply a magnetic field for precipitation, remove the supernatant, wash the precipitated complex with washing solution 3 times, and enter the sample measurement chamber. The instrument (automatic chemiluminescence immunoassay analyzer MAGLUMI X8) was automatically pumped into the automatic immunoassay system with substrates 1 and 2, and the relative light intensity (RLU) emitted within 3 sec was automatically monitored. LN concentration was proportional to RLU, and the detection instrument automatically fitted and calculated LN concentration. The kits were provided by New Industry Biomedical Engineering Co., LTD (Shenzhen, China).

### Follow-up

The primary endpoint was MACE, which mainly included composite death, recurrent myocardial infarction, and heart failure hospitalization within 6 months. Endpoint data were obtained from the hospital database and telephone follow-ups, which were validated by reviewing medical records. We completed 100% of the follow-up visits.

### Statistical analysis

The measurement data that conformed to the normal distribution are described in the form of mean ± standard deviation, and the independent sample *T*-test was used for statistical analysis. Data that did not conform to the normal distribution were described using the M-estimator (P25, P75) and the non-parametric test (Kruskal-Wallis test) for statistical analysis. The Chi-square test was used for categorical variables. Independent predictors of myocardial infarction prognosis were screened using logistic regression analysis. Finally, the receiver operating characteristic (ROC) curve was used to judge the predictive value of LN for the occurrence of MACE. All statistical analyses were performed using SPSS version 23 (IBM SPSS Statistics, IBM Corporation Armonk, New York).

## Results

### Comparison of baseline data between the two groups

Univariate analysis showed that there was no significant difference in gender, Hypertension, Diabetes, BMI, Ccr, ALT, AST, LDL-C between the two groups (*p* > 0.05). Smoking (%), age, N-terminal prohormone brain natriuretic peptide (NT-proBNP), cardiac troponin I (c-TNI), and LN were significantly higher in the MACE group than in the non-MACE group (*p* < 0.05). Conversely, emergency percutaneous coronary intervention (%) was significantly lower in the MACE group than in the non-MACE group (*p* < 0.05) (Table 1).

**Table 1:** Comparison of baseline data between two groups.

	**MACE group(*****n*** = **47)**	**Non-MACE group (*****n*** = **155)**	**p**
Man *(n (%)*	32(68.09)	120(77.42)	0.194
Hypertension *(n (%)*	26(55.32)	92(59.35)	0.623
Diabetes *(n (%)*	22(46.81)	54(34.84)	0.138
Smoking *(n (%)*	30(63.83)	72(46.45)	0.037
EPCI *(n (%)*	18(38.30)	115(74.19)	<0.001
Age (years)	73.79 ± 12.36	65.64 ± 12.80	<0.001
BMI (kg/m^2^)	29.76 ± 9.40	30.03 ± 9.81	0.868
Ccr (ml/min/1.73 m^2^)	77.11 ± 36.20	88.22 ± 35.09	0.061
c-TNI (ug/L)	31.60(3.54, 60.03)	5.16 (0.31, 31.70)	0.001
NT-proBNP (pg/ml)	4971.00(2363.00, 10321.00)	1405.00(482.50, 4093.00)	<0.001
ALT (U/L)	35.00(22.00, 98.00)	40.00(22.00, 129.00)	0.609
AST (U/L)	25.00(16.00, 48.00)	28.00(18.00, 50.00)	0.827
LDL-C(mmol/L)	4.45(3.55, 5.25)	4.49(3.35, 5.32)	0.912
LN (ng/ml)	105.20(74.46, 157.00)	31.85(22.67, 56.03)	<0.001

*EPCI, emergency percutaneous coronary intervention; BMI, Body Mass Index; Ccr, Creatinine Clearance; c-TNI, cardiac troponin I; NT-proBNP, N-terminal B-type natriuretic peptide precursor; ALT, alanine amiotransferase; AST, aspartate transaminase; LN, Laminin*.

*c-TNI/ NT-proBNP/ ALT/ AST/LDL-C/LN did not conform to the normal distribution and was described in the form of M (P25, P75)*.

### Multivariate logistic regression of MACE in patients with AMI

Taking the occurrence of MACE as a dependent variable and the related factors (smoking, EPCI, age, c-TNI, NT-proBNP, LN) as independent variables in univariate analysis, a multivariate logistic regression analysis was performed. Multivariate regression analysis demonstrated that the level of LN was an independent predictor of MACE 6 months after a myocardial infarction [OR = 1.022, 95% CI = (1.014, 1.032), *p* < 0.001]. In addition, age [OR = 1.074, 95% CI = (1.030, 1.119), *p* = 0.001] and EPCI [OR = 0.215, 95% CI = (0.087, 0.526), *p* = 0.001] were independently associated with MACE after 6 months (Table 2).

**Table 2:** Binary logistic regression analysis of independent predictors of MACE.

**Characteristics**	**OR**	**95%CI**		**p**
LN	1.022	1.014	1.032	<0.001
Age	1.074	1.030	1.119	0.001
Smoking	2.000	0.791	5.051	0.143
c-TNI	1.025	1.008	1.041	0.003
NT-proBNP	1.000	1.000	1.000	0.398
EPCI	0.215	0.087	0.526	0.001

*LN, Laminin; c-TNI: cardiac troponin I; NT-proBNP, N-terminal B-type natriuretic peptide precursor; EPCI, emergency percutaneous coronary intervention*.

### Predictive value of LN for the risk of MACE in AMI patients

**Figure 1:**
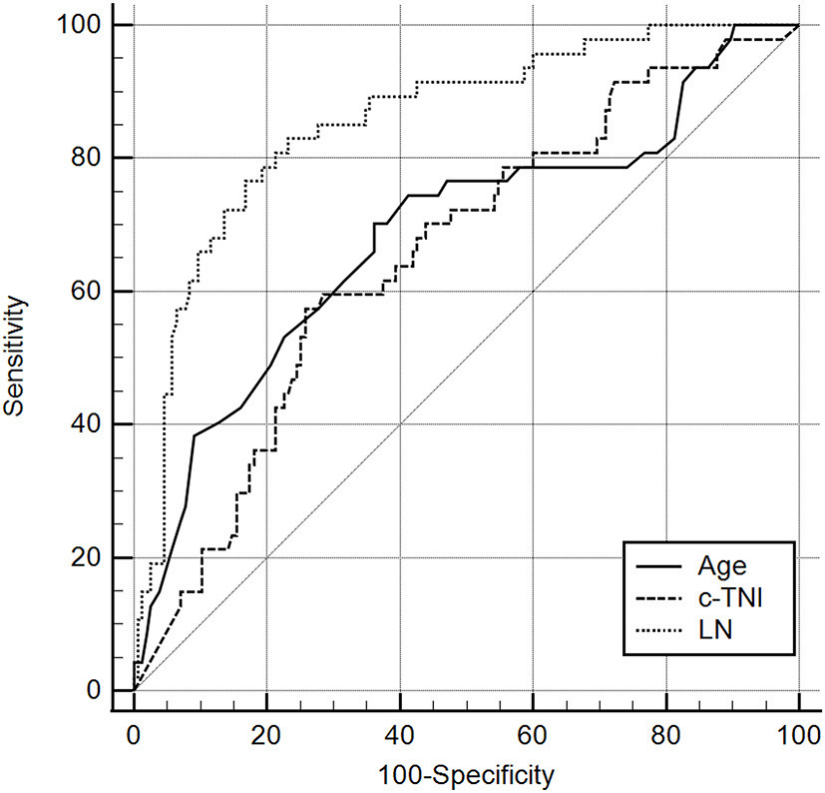
Receiver operating characteristic curves for laminin, age, and c-TNI in the prediction of MACE in AMI patients. The results showed that the predictive value of LN was significantly higher than that of Age (AUC 0.856 > 0.686, *P* = 0.0066 <0.05). It was also significantly higher than that of C-TNI (AUC 0.856 > 0.0655, *P* = 0.0007 <0.05).

The area under the ROC curve of LN for predicting the occurrence of MACE was 0.856 [95% CI = (0.800–0.901)], and a cut-off value of 58.80 ng/ml (sensitivity = 83.00%, specificity = 76.80%) or 74.15 ng/ml (sensitivity = 76.6%, specificity = 83.2%) indicate a poor prognosis for AMI (Different cut-off values are selected according to the need for higher sensitivity or specificity in clinical applications). Compared with c-TNI (AUC = 0.655 < 0.856, *p* = 0.0007) and age (AUC = 0.686 < 0.856, *p* = 0.0066), LN had a better predictive value for MACE in patients with AMI (Figure 1; Tables 3, 4).

**Table 3:** Receiver operating characteristic curves for laminin, age, and c-TNI in the prediction of MACE in AMI patients.

**Variable**	**AUC**	**SE**	**95%CI**	
Age	0.686	0.0481	0.617	0.749
c-TNI	0.655	0.0450	0.585	0.720
LN	0.856	0.0316	0.800	0.901

*LN, Laminin; c-TNI, cardiac troponin I*.

**Table 4:** Pairwise comparison of ROC curves for laminin, age, and c-TNI in the prediction of MACE in AMI patients.

**Pairwise comparison**	***p*** **-value**
Age ~ LN	0.0066
c-TNI ~ LN	0.0007

*LN, Laminin; c-TNI, cardiac troponin I*.

## Discussion

The myocardial ECM is composed of collagen, fibronectin (FN), LN, elastin, and proteoglycans. In response to ischemic injury, neurohormonal activation, or changes in hemodynamic load, the ECM undergoes structural remodeling, involving collagen degeneration, production of new collagen, changes in the ratio of collagen phenotypes, and changes in the amount of collagen cross-linking (10). Studies have confirmed that ventricular remodeling is a key factor leading to a poor prognosis in acute myocardial infarction, and that myocardial fibrosis is the main pathological manifestation of myocardial remodeling (11). In the pathological condition of myocardial infarction, myocardial fibroblasts are the main cells tasked to produce ECM (3). Changes in its quantity and function are also associated with extracellular matrix deposition and fibrosis in various tissues and organs (the liver and kidney). Increased deposition of myocardial ECM and changes in collagen composition are closely related to myocardial fibrosis, which in turn is an important factor affecting cardiac function (3, 4, 12). Many studies have suggested that serum levels of extracellular matrix proteins (or their cleavage fragments) may be used to assess the severity and progression of myocardial remodeling (13–15). Changes in the myocardial cytoskeleton and extracellular matrix may affect systolic function because the cytoskeleton organizes the intracellular and intercellular structures. Understanding the basic mechanisms that regulate injury responses is critical for the development of site-specific cell biological intervention strategies to reduce injury and promote repair (10).

At present, numerous studies have found that many markers of liver fibrosis, such as N-terminal propeptide of procollagen III (PIIINP), 7S domain of the collagen type IV N-terminal propeptide, (P4NP7S) (16), hyaluronic acid (HA) (17) can also be used as indicators of myocardial fibrosis. Laminin is also one of the indicators of liver fibrosis.

Laminin is a non-collagen glycoprotein first discovered by Timple et al. (18). Laminins belong to a family of 16 distinct heterotrimeric proteins. Each laminin isomer is composed of α, β and γ Chain composition, each named after a numerical subtype. Lamellae are widespread but often overlap. Each laminin has a unique phenotype that affects the differentiation and/or maintenance of different tissues from the earliest stages of embryogenesis to adulthood.

Thus far, studies on changes in parameters reflecting the integrity of the basement membrane in patients with acute myocardial infarction have not been sufficient. We can prove that the increase in LN levels after AMI can predict the occurrence of short-term MACE (6 months after an acute myocardial infarction), which may be a substitute marker for the early activation of extracellular myocardial matrix metabolism. In the early stages of AMI, this rapid activation of LN may represent non-specific repair because it is independent of other clinical variables.

The increase in serum LN levels in the early stages of myocardial infarction can be explained by the biological function of the LN family. Laminin is a major structural component of the basement membrane and plays a role in cell proliferation, adhesion, differentiation and migration (10).

Elevated serum LN antigen concentrations have been reported in patients with liver fibrosis and cirrhosis, regardless of the source of the disease (alcohol abuse or chronic viral hepatitis B or C infection) (19–22). Furthermore, LN concentration is associated with the degree of fibrosis and the grade of hepatic fibrosis (23). Animal studies have shown that (24) a large amount of LN is present in the myocardial interstitium of hypertensive rats. Furthermore, LN contributes to extracellular matrix assembly during early healing after myocardial infarction in rats (25). In 2009, Dinh et al. found that the elevation of serum laminin levels in patients with acute myocardial infarction suggested early myocardial remodeling and predicted the progression of myocardial fibrosis (10). A report in 2020 combined with previous basic studies found that laminin-α5 is upregulated transiently in the basement membrane in human and murine muscular dystrophy which is accompanied by cardiomyopathy (26). Laminin can reflect the activity and proliferation of fibroblasts and can be used as an indicator of myocardial fibrosis detection; to a certain extent, it reflects the degree of myocardial tissue damage and the pathological process of secondary myocardial fibrosis (27, 28).

In this study, we noted that laminin levels were significantly higher in patients with AMI who had MACE than in those without MACE. We speculate that laminin reflects early extracellular matrix remodeling and is involved in tissue repair after ischemia. As an indicator of liver fibrosis, laminin could also suggest the occurrence of myocardial fibrosis. More importantly, laminin may be of greater value in the future treatment of AMI patients to improve prognosis.

Our study has some limitations. The progression of remodeling is influenced by many different factors, such as metalloproteinase (MMP) and metalloproteinase tissue inhibitor (TIMP) systems, cytokines, and neuroendocrine activation (29). In addition, the ECM structure varies from patient to patient, in terms of the number of myocytes and fibroblasts. Laminin levels may depend not only on the magnitude of acute injury but also on other pathophysiological changes initiated after AMI. Therefore, the degree of LN elevation does not fully reflect the magnitude of acute myocardial injury or degree of left ventricular remodeling. Rather, it reflects the ability of the collagen matrix to degrade during remodeling, as part of a complex regulatory system.

In conclusion, our findings suggest that the development of short-term MACE due to early cardiac remodeling after ischemic injury is reflected by an increase in serum LN levels. Therefore, LN may be a predictor of MACE following AMI. It is also necessary to expand the sample size and extend the follow-up time to further demonstrate the correlation between LN level and AMI prognosis.

## Data availability statement

The original contributions presented in the study are included in the article/supplementary material, further inquiries can be directed to the corresponding author.

## Ethics statement

Written informed consent was obtained from the individual(s) for the publication of any potentially identifiable images or data included in this article.

## Author contributions

L-YX: conceptualization, methodology, formal analysis, data curation, resources, writing—original draft, writing—review and editing, project administration, and funding acquisition. LX: investigation, validation, formal analysis, data curation, writing, and original draft. JW: formal analysis and investigation. H-XC: formal analysis and data curation. H-LC: formal analysis and validation. L-JT and QZ: resources, writing—review and editing, supervision, project administration, and funding acquisition. All authors contributed to the article and approved the submitted version.

## Funding

This work was supported by the Scientific Research Project of Nantong Municipal Health Commission (grant MB2021010), Kangda College of Nanjing Medical University (grant KD2021KYJJZD012), and Nantong Science and Technology Bureau Plan Project (grant JC2020054).

## Conflict of interest

The authors declare that the research was conducted in the absence of any commercial or financial relationships that could be construed as a potential conflict of interest.

## Publisher's note

All claims expressed in this article are solely those of the authors and do not necessarily represent those of their affiliated organizations, or those of the publisher, the editors and the reviewers. Any product that may be evaluated in this article, or claim that may be made by its manufacturer, is not guaranteed or endorsed by the publisher.
