# Supplementary material for: Correlation between serum laminin levels and prognosis of acute myocardial infarction
Source: Front Cardiovasc Med. 2022 Jul 22;9:936983. doi: 10.3389/fcvm.2022.936983 (PMC9363112; doi:10.3389/fcvm.2022.936983)
Supplement: Supplementary file 1 [file Data_Sheet_1.PDF]

| order number | LN | sex | age | HBP | DM | height | weight | BMI | smoking | ALT | AST | CTNI | BNP |
|---|---|---|---|---|---|---|---|---|---|---|---|---|---|
| 1 | 21.62 | 2 | 78 | 1 | 1 | 148 | 47.4 | 15.18081 | 2 | 11 | 17 | 40.15 | 201.7 |
| 2 | 24.2 | 1 | 86 | 1 | 2 | 170 | 70 | 28.82353 | 1 | 22 | 49 | 38.5 | 2042 |
| 3 | 28.15 | 2 | 73 | 1 | 2 | 160 | 71 | 31.50625 | 1 | 37 | 26 | 3.05 | 139.8 |
| 4 | 28.34 | 1 | 81 | 1 | 2 | 166 | 65 | 25.45181 | 1 | 50 | 268 | 78.1 | 5662 |
| 5 | 35.7 | 1 | 91 | 1 | 2 | 165 | 65 | 25.60606 | 1 | 23 | 38 | 9 | 12590 |
| 6 | 40.67 | 1 | 84 | 2 | 2 | 170 | 77.2 | 35.05788 | 1 | 16 | 22 | 60.03 | 4682 |
| 7 | 44.2 | 1 | 81 | 1 | 2 | 180 | 89 | 44.00556 | 1 | 14 | 22 | 80 | 2962 |
| 8 | 51.23 | 1 | 80 | 1 | 1 | 172 | 80 | 37.2093 | 1 | 35 | 28 | 72.5 | 5000 |
| 9 | 58.9 | 1 | 71 | 2 | 2 | 170 | 60 | 21.17647 | 1 | 38 | 35 | 59.09 | 351.7 |
| 10 | 64.19 | 2 | 78 | 1 | 1 | 155 | 69 | 30.71613 | 1 | 59 | 50 | 80 | 8799 |
| 11 | 69.1 | 1 | 63 | 2 | 2 | 117 | 77 | 50.67521 | 2 | 41 | 154 | 35 | 4390 |
| 12 | 74.46 | 2 | 95 | 2 | 2 | 155 | 50 | 16.12903 | 2 | 19 | 35 | 33.63 | 6855 |
| 13 | 74.93 | 1 | 84 | 2 | 1 | 155 | 68 | 29.83226 | 1 | 6 | 9 | 50.13 | 2531 |
| 14 | 80.01 | 1 | 79 | 1 | 1 | 176 | 70 | 27.84091 | 2 | 20 | 28 | 80 | 5237 |
| 15 | 80.4 | 2 | 83 | 2 | 2 | 160 | 70 | 30.625 | 1 | 48 | 35 | 69 | 19221 |
| 16 | 86.29 | 2 | 71 | 1 | 1 | 150 | 74 | 36.50667 | 1 | 13 | 27 | 3.54 | 10321 |
| 17 | 91.03 | 2 | 81 | 1 | 2 | 182 | 69 | 26.15934 | 2 | 44 | 81 | 37 | 11153 |
| 18 | 91.73 | 2 | 81 | 1 | 1 | 156 | 88 | 49.64103 | 2 | 103 | 96 | 12.6 | 3671 |
| 19 | 96.16 | 2 | 50 | 2 | 1 | 160 | 65 | 26.40625 | 1 | 38 | 225 | 70 | 4698 |
| 20 | 101.6 | 1 | 76 | 2 | 1 | 178 | 88 | 43.50562 | 1 | 93 | 369 | 80 | 5235 |
| 21 | 103.2 | 1 | 54 | 1 | 2 | 158 | 60 | 22.78481 | 1 | 93 | 98 | 18.8 | 2654 |
| 22 | 103.4 | 1 | 74 | 1 | 1 | 175 | 64 | 23.40571 | 2 | 19 | 35 | 0.63 | 5363 |
| 23 | 105.2 | 1 | 78 | 1 | 2 | 178 | 72 | 29.1236 | 1 | 9 | 18 | 6.81 | 2248 |
| 24 | 105.2 | 2 | 83 | 1 | 1 | 155 | 45 | 13.06452 | 2 | 29 | 169 | 64.1 | 423.9 |
| 25 | 113.3 | 2 | 54 | 2 | 1 | 163 | 63 | 24.34969 | 1 | 47 | 177 | 80 | 2363 |
| 26 | 123.9 | 1 | 81 | 2 | 1 | 163 | 65 | 25.92025 | 2 | 36 | 26 | 80 | 16396 |
| 27 | 129.2 | 1 | 54 | 1 | 1 | 178 | 87.5 | 43.01264 | 2 | 46 | 119 | 53.2 | 7274 |
| 28 | 135.8 | 1 | 57 | 2 | 1 | 171 | 73 | 31.16374 | 1 | 59 | 67 | 14 | 1312 |
| 29 | 137.7 | 1 | 87 | 2 | 2 | 174 | 90 | 46.55172 | 1 | 80 | 99 | 21.9 | 35000 |
| 30 | 140.3 | 1 | 74 | 2 | 1 | 188 | 86 | 39.34043 | 1 | 171 | 375 | 80 | 4077 |
| 31 | 141 | 1 | 55 | 2 | 2 | 165 | 70 | 29.69697 | 1 | 56 | 61 | 31.6 | 2069 |
| 32 | 141.8 | 1 | 82 | 1 | 2 | 172 | 78 | 35.37209 | 2 | 9 | 12 | 29 | 35000 |
| 33 | 149.7 | 1 | 86 | 1 | 1 | 165 | 55 | 18.33333 | 2 | 25 | 121 | 59.9 | 16595 |
| 34 | 153.3 | 1 | 76 | 2 | 2 | 172 | 81 | 38.14535 | 1 | 13 | 19 | 0.08 | 6053 |
| 35 | 156.6 | 2 | 87 | 2 | 1 | 158 | 58 | 21.29114 | 2 | 12 | 15 | 0.01 | 3969 |
| 36 | 157 | 1 | 49 | 2 | 1 | 170 | 70 | 28.82353 | 1 | 21 | 33 | 47.2 | 1379 |

| | | | | | | | | | | | | | |
|---|---|---|---|---|---|---|---|---|---|---|---|---|---|
| 37 | 159.8 | 1 | 73 | 2 | 1 | 168 | 74 | 32.59524 | 2 | 18 | 20 | 42 | 23597 |
| 38 | 164.2 | 1 | 75 | 2 | 2 | 170 | 80 | 37.64706 | 2 | 61 | 65 | 26 | 25258 |
| 39 | 173.8 | 2 | 85 | 1 | 2 | 178 | 79 | 35.0618 | 2 | 6 | 15 | 0.06 | 7868 |
| 40 | 183.1 | 2 | 84 | 2 | 2 | 155 | 33 | 7.025806 | 1 | 18 | 22 | 29 | 35000 |
| 41 | 205 | 1 | 77 | 1 | 2 | 165 | 70 | 29.69697 | 1 | 23 | 17 | 0.78 | 1487 |
| 42 | 213.6 | 1 | 50 | 2 | 2 | 169 | 75 | 33.28402 | 1 | 43 | 161 | 31.5 | 793.3 |
| 43 | 230.6 | 1 | 53 | 1 | 1 | 169 | 55 | 17.89941 | 1 | 55 | 44 | 0.47 | 2512 |
| 44 | 254.5 | 1 | 77 | 1 | 1 | 175 | 88 | 44.25143 | 1 | 19 | 23 | 0.2 | 5273 |
| 45 | 268.7 | 2 | 68 | 1 | 2 | 165 | 60 | 21.81818 | 1 | 21 | 25 | 2.34 | 2685 |
| 46 | 320.4 | 1 | 54 | 1 | 2 | 170 | 70 | 28.82353 | 1 | 16 | 73 | 1.07 | 35000 |
| 47 | 371.3 | 1 | 76 | 1 | 2 | 170 | 70 | 28.82353 | 2 | 14 | 29 | 3.81 | 8150 |
| 48 | 11.82 | 1 | 49 | 2 | 2 | 172 | 69 | 27.68023 | 1 | 77 | 487 | 10 | 1835 |
| 49 | 12.95 | 1 | 60 | 1 | 2 | 174 | 80 | 36.78161 | 1 | 62 | 157 | 5.16 | 1101 |
| 50 | 13.23 | 1 | 67 | 1 | 1 | 170 | 76 | 33.97647 | 1 | 50 | 26 | 0.13 | 313.6 |
| 51 | 13.96 | 2 | 57 | 1 | 2 | 160 | 62 | 24.025 | 2 | 15 | 37 | 2.49 | 233.8 |
| 52 | 14.1 | 1 | 76 | 1 | 2 | 165 | 63 | 24.05455 | 2 | 21 | 30 | 28 | 7011 |
| 53 | 14.67 | 1 | 56 | 2 | 2 | 168 | 73 | 31.72024 | 2 | 19 | 22 | 0.16 | 796.4 |
| 54 | 14.9 | 1 | 62 | 2 | 2 | 168 | 58 | 20.02381 | 1 | 21 | 26 | 1.4 | 1227 |
| 55 | 15 | 1 | 83 | 2 | 2 | 165 | 55 | 18.33333 | 2 | 10 | 72 | 26 | 5762 |
| 56 | 15.11 | 2 | 59 | 2 | 2 | 162 | 54 | 18 | 2 | 47 | 32 | 0.26 | 1726 |
| 57 | 15.27 | 1 | 70 | 2 | 1 | 166 | 72 | 31.22892 | 2 | 23 | 31 | 0.83 | 937.4 |
| 58 | 15.28 | 1 | 30 | 2 | 2 | 160 | 55 | 18.90625 | 2 | 46 | 116 | 22.4 | 157.9 |
| 59 | 16.01 | 1 | 75 | 2 | 2 | 165 | 55 | 18.33333 | 1 | 216 | 24 | 0.18 | 3302 |
| 60 | 16.33 | 2 | 75 | 1 | 2 | 159 | 73.2 | 33.69962 | 1 | 13 | 17 | 0.04 | 872 |
| 61 | 16.45 | 1 | 37 | 2 | 2 | 157 | 84 | 44.94268 | 1 | 108 | 369 | 43 | 618.8 |
| 62 | 16.69 | 1 | 69 | 1 | 2 | 180 | 67 | 24.93889 | 2 | 50 | 34 | 1.43 | 4333 |
| 63 | 17.24 | 1 | 61 | 1 | 1 | 175 | 78 | 34.76571 | 1 | 13 | 16 | 0.02 | 100.4 |
| 64 | 17.36 | 1 | 72 | 2 | 2 | 182 | 75.5 | 31.32005 | 1 | 16 | 20 | 0.03 | 38 |
| 65 | 17.71 | 1 | 53 | 2 | 2 | 170 | 68.5 | 27.60147 | 1 | 21 | 18 | 0.06 | 313.8 |
| 66 | 18.33 | 1 | 81 | 2 | 2 | 175 | 86.7 | 42.95366 | 2 | 21 | 22 | 1.64 | 162.8 |
| 67 | 18.44 | 1 | 69 | 1 | 1 | 175 | 85 | 41.28571 | 1 | 18 | 16 | 2.76 | 112.3 |
| 68 | 18.98 | 2 | 69 | 2 | 1 | 162 | 50 | 15.4321 | 2 | 9 | 20 | 0.13 | 35000 |
| 69 | 19.15 | 1 | 75 | 2 | 2 | 168 | 78 | 36.21429 | 1 | 10 | 14 | 0.03 | 126.6 |
| 70 | 19.22 | 1 | 60 | 1 | 2 | 172 | 90 | 47.09302 | 2 | 33 | 22 | 0.11 | 70.5 |
| 71 | 19.96 | 1 | 57 | 1 | 2 | 162 | 75 | 34.72222 | 2 | 22 | 32 | 2.91 | 782.8 |
| 72 | 20.16 | 1 | 50 | 1 | 2 | 170 | 78 | 35.78824 | 2 | 8 | 15 | 2.83 | 965.6 |
| 73 | 20.17 | 1 | 62 | 2 | 2 | 166 | 53.9 | 17.50127 | 2 | 11 | 13 | 0.06 | 45.1 |
| 74 | 20.26 | 2 | 81 | 1 | 1 | 156 | 58 | 21.5641 | 2 | 9 | 19 | 0.02 | 5847 |

| | | | | | | | | | | | | | |
|---|---|---|---|---|---|---|---|---|---|---|---|---|---|
| 75 | 20.3 | 1 | 58 | 1 | 2 | 170 | 77 | 34.87647 | 2 | 14 | 16 | 0.47 | 788.9 |
| 76 | 20.41 | 1 | 75 | 1 | 2 | 170 | 60 | 21.17647 | 1 | 28 | 102 | 25.3 | 1623 |
| 77 | 20.67 | 1 | 79 | 1 | 1 | 168 | 83 | 41.00595 | 2 | 50 | 26 | 3.31 | 2175 |
| 78 | 20.9 | 1 | 78 | 2 | 2 | 160 | 77 | 37.05625 | 2 | 31 | 23 | 45.4 | 8280 |
| 79 | 21.33 | 1 | 60 | 1 | 1 | 181 | 80 | 35.35912 | 1 | 35 | 140 | 8.86 | 453.6 |
| 80 | 21.34 | 1 | 50 | 2 | 2 | 175 | 65 | 24.14286 | 2 | 59 | 249 | 80 | 383.7 |
| 81 | 21.37 | 2 | 48 | 2 | 2 | 170 | 60 | 21.17647 | 1 | 11 | 15 | 0.05 | 132.9 |
| 82 | 21.38 | 1 | 74 | 1 | 2 | 174 | 67.2 | 25.9531 | 2 | 26 | 23 | 0.02 | 133.1 |
| 83 | 21.82 | 1 | 67 | 1 | 2 | 155 | 60 | 23.22581 | 2 | 43 | 158 | 25.7 | 475.4 |
| 84 | 22.11 | 1 | 63 | 2 | 1 | 176 | 72 | 29.45455 | 1 | 50 | 182 | 23.3 | 461.9 |
| 85 | 22.52 | 1 | 63 | 1 | 2 | 170 | 70 | 28.82353 | 2 | 38 | 178 | 24.4 | 1071 |
| 86 | 22.67 | 1 | 56 | 1 | 2 | 172 | 95 | 52.47093 | 1 | 60 | 253 | 1.79 | 433.9 |
| 87 | 22.7 | 2 | 62 | 2 | 2 | 160 | 60 | 22.5 | 2 | 35 | 25 | 23.8 | 3030 |
| 88 | 22.8 | 2 | 74 | 1 | 2 | 160 | 65 | 26.40625 | 2 | 23 | 136 | 1.08 | 2147 |
| 89 | 22.88 | 2 | 68 | 2 | 2 | 162 | 65 | 26.08025 | 2 | 19 | 14 | 0.15 | 7.3 |
| 90 | 22.9 | 1 | 65 | 2 | 2 | 170 | 60 | 21.17647 | 2 | 22 | 27 | 4.63 | 967.5 |
| 91 | 23 | 1 | 71 | 1 | 2 | 170 | 52.5 | 16.21324 | 2 | 10 | 22 | 1.37 | 1212 |
| 92 | 23.05 | 1 | 71 | 1 | 1 | 170 | 69 | 28.00588 | 1 | 15 | 20 | 0.31 | 791.1 |
| 93 | 23.19 | 2 | 78 | 1 | 1 | 153 | 75 | 36.76471 | 2 | 11 | 15 | 1.78 | 873.2 |
| 94 | 23.49 | 1 | 55 | 2 | 2 | 170 | 70 | 28.82353 | 1 | 34 | 81 | 5.23 | 188.1 |
| 95 | 23.61 | 2 | 55 | 1 | 1 | 157 | 81 | 41.78981 | 2 | 14 | 16 | 0.01 | 1422 |
| 96 | 23.7 | 1 | 62 | 2 | 2 | 174 | 67 | 25.79885 | 2 | 27 | 22 | 0.36 | 49.6 |
| 97 | 23.78 | 1 | 78 | 2 | 2 | 165 | 65 | 25.60606 | 1 | 44 | 269 | 34.1 | 984.4 |
| 98 | 24.53 | 1 | 67 | 1 | 2 | 160 | 65 | 26.40625 | 2 | 36 | 129 | 4.79 | 1213 |
| 99 | 24.7 | 1 | 64 | 1 | 1 | 168 | 88 | 46.09524 | 1 | 23 | 60 | 29.5 | 1405 |
| 100 | 24.81 | 1 | 50 | 1 | 1 | 165 | 70 | 29.69697 | 1 | 93 | 320 | 80 | 1990 |
| 101 | 25 | 1 | 50 | 2 | 2 | 180 | 76.7 | 32.68272 | 2 | 21 | 21 | 0.1 | 958.5 |
| 102 | 25 | 2 | 85 | 1 | 2 | 150 | 82 | 44.82667 | 2 | 12 | 23 | 7 | 13819 |
| 103 | 25.13 | 1 | 76 | 1 | 2 | 156 | 70 | 31.41026 | 2 | 37 | 110 | 9.96 | 1073 |
| 104 | 25.22 | 1 | 71 | 2 | 2 | 165 | 68 | 28.02424 | 1 | 26 | 132 | 58.4 | 813.7 |
| 105 | 25.55 | 1 | 61 | 2 | 1 | 153 | 61 | 24.32026 | 2 | 45 | 146 | 3.9 | 384.1 |
| 106 | 25.56 | 1 | 77 | 1 | 1 | 190 | 89.7 | 42.34784 | 1 | 22 | 54 | 1.65 | 6180 |
| 107 | 25.6 | 1 | 54 | 1 | 2 | 173 | 70 | 28.3237 | 2 | 68 | 234 | 18.9 | 419.8 |
| 108 | 25.83 | 1 | 66 | 1 | 2 | 169 | 65 | 25 | 1 | 20 | 43 | 2.59 | 715.9 |
| 109 | 27.67 | 1 | 57 | 2 | 2 | 168 | 86 | 44.02381 | 1 | 53 | 200 | 77 | 48.5 |
| 110 | 28.2 | 1 | 75 | 1 | 2 | 167 | 70 | 29.34132 | 2 | 18 | 29 | 2.78 | 2189 |
| 111 | 28.3 | 1 | 62 | 1 | 2 | 170 | 70 | 28.82353 | 2 | 42 | 175 | 25 | 487.5 |
| 112 | 28.35 | 1 | 76 | 2 | 2 | 185 | 70 | 26.48649 | 1 | 10 | 28 | 2.33 | 1253 |

| | | | | | | | | | | | | | |
|---|---|---|---|---|---|---|---|---|---|---|---|---|---|
| 113 | 28.71 | 1 | 77 | 1 | 2 | 182 | 84 | 38.76923 | 1 | 14 | 44 | 4.49 | 639.3 |
| 114 | 29.1 | 1 | 59 | 1 | 2 | 170 | 69 | 28.00588 | 2 | 27 | 35 | 2.91 | 163.5 |
| 115 | 29.29 | 1 | 70 | 2 | 2 | 164 | 65 | 25.7622 | 2 | 6 | 15 | 1.46 | 3412 |
| 116 | 29.3 | 1 | 53 | 2 | 2 | 172 | 72 | 30.13953 | 2 | 23 | 17 | 4.9 | 2722 |
| 117 | 29.34 | 2 | 70 | 2 | 2 | 163 | 63 | 24.34969 | 1 | 19 | 54 | 13.7 | 750.9 |
| 118 | 29.47 | 2 | 68 | 1 | 2 | 156 | 57.2 | 20.97333 | 2 | 12 | 14 | 0.12 | 384.3 |
| 119 | 29.6 | 2 | 42 | 2 | 1 | 155 | 65 | 27.25806 | 2 | 34 | 37 | 0.11 | 1686 |
| 120 | 29.79 | 1 | 45 | 1 | 2 | 170 | 74 | 32.21176 | 2 | 54 | 198 | 27.7 | 212.9 |
| 121 | 30.22 | 1 | 41 | 2 | 2 | 175 | 90 | 46.28571 | 2 | 66 | 102 | 31.7 | 390 |
| 122 | 30.45 | 1 | 43 | 1 | 2 | 166 | 100 | 60.24096 | 1 | 104 | 221 | 80 | 789.1 |
| 123 | 31.06 | 1 | 38 | 2 | 2 | 170 | 75 | 33.08824 | 1 | 111 | 384 | 77.6 | 1112 |
| 124 | 31.21 | 1 | 61 | 1 | 2 | 168 | 80 | 38.09524 | 2 | 30 | 108 | 6.89 | 3070 |
| 125 | 31.85 | 1 | 61 | 1 | 1 | 184 | 63 | 21.57065 | 2 | 171 | 44 | 80 | 464 |
| 126 | 32.17 | 1 | 51 | 1 | 2 | 175 | 75 | 32.14286 | 1 | 30 | 22 | 80 | 134.4 |
| 127 | 32.57 | 1 | 75 | 1 | 2 | 172 | 70 | 28.48837 | 1 | 15 | 25 | 0.05 | 4766 |
| 128 | 32.74 | 1 | 37 | 2 | 2 | 177 | 70 | 27.68362 | 1 | 108 | 369 | 43 | 618.8 |
| 129 | 33.59 | 1 | 74 | 1 | 1 | 178 | 80.3 | 36.22522 | 1 | 14 | 44 | 0.01 | 3135 |
| 130 | 33.83 | 1 | 66 | 1 | 2 | 176 | 80 | 36.36364 | 2 | 53 | 225 | 0.23 | 726.7 |
| 131 | 33.86 | 1 | 55 | 1 | 1 | 175 | 65 | 24.14286 | 2 | 23 | 98 | 20.2 | 1153 |
| 132 | 34.12 | 1 | 76 | 1 | 2 | 174 | 80 | 36.78161 | 2 | 19 | 50 | 17.7 | 1283 |
| 133 | 34.32 | 2 | 80 | 1 | 2 | 156 | 52.4 | 17.60103 | 2 | 18 | 12 | 0.94 | 372.1 |
| 134 | 34.4 | 2 | 62 | 1 | 2 | 160 | 60 | 22.5 | 2 | 43 | 112 | 23.8 | 3032 |
| 135 | 35.13 | 1 | 57 | 1 | 1 | 161 | 75 | 34.93789 | 2 | 26 | 21 | 0.04 | 2816 |
| 136 | 35.16 | 1 | 79 | 1 | 2 | 165 | 60 | 21.81818 | 2 | 35 | 164 | 66.1 | 1363 |
| 137 | 35.9 | 2 | 67 | 1 | 1 | 150 | 55 | 20.16667 | 1 | 17 | 27 | 1.23 | 6614 |
| 138 | 36 | 1 | 60 | 1 | 2 | 180 | 85 | 40.13889 | 2 | 12 | 76 | 5.07 | 3952 |
| 139 | 36.41 | 2 | 83 | 1 | 1 | 155 | 45 | 13.06452 | 1 | 29 | 169 | 64.1 | 423.9 |
| 140 | 36.57 | 1 | 53 | 2 | 2 | 175 | 75 | 32.14286 | 1 | 51 | 257 | 68.3 | 967.5 |
| 141 | 37.4 | 1 | 65 | 2 | 2 | 175 | 78 | 34.76571 | 2 | 75 | 311 | 23.2 | 5675 |
| 142 | 38.2 | 1 | 65 | 1 | 2 | 176 | 68 | 26.27273 | 2 | 53 | 210 | 37.1 | 1879 |
| 143 | 38.6 | 1 | 76 | 2 | 2 | 163 | 69 | 29.20859 | 1 | 25 | 34 | 3.8 | 22220.9 |
| 144 | 39.57 | 1 | 46 | 2 | 2 | 180 | 65 | 23.47222 | 1 | 16 | 30 | 0.29 | 940 |
| 145 | 39.89 | 1 | 69 | 2 | 2 | 170 | 70 | 28.82353 | 1 | 100 | 228 | 80 | 482.5 |
| 146 | 40.24 | 1 | 54 | 1 | 2 | 160 | 63 | 24.80625 | 1 | 49 | 44 | 8.14 | 1039 |
| 147 | 40.32 | 2 | 80 | 1 | 1 | 170 | 75 | 33.08824 | 1 | 31 | 27 | 0.16 | 323.9 |
| 148 | 44.06 | 2 | 58 | 2 | 2 | 163 | 75 | 34.5092 | 2 | 22 | 67 | 15.6 | 1973 |
| 149 | 44.5 | 1 | 40 | 1 | 2 | 178 | 62 | 21.59551 | 1 | 34 | 67 | 8.73 | 639.3 |
| 150 | 44.94 | 1 | 80 | 2 | 2 | 158 | 53.3 | 17.98032 | 1 | 21 | 19 | 0.17 | 131 |

| | | | | | | | | | | | | | |
|---|---|---|---|---|---|---|---|---|---|---|---|---|---|
| 151 | 45.26 | 2 | 56 | 1 | 2 | 162 | 65 | 26.08025 | 1 | 43 | 105 | 42.5 | 3643 |
| 152 | 45.3 | 1 | 79 | 1 | 1 | 169 | 50 | 14.7929 | 1 | 33 | 94 | 6.32 | 4253 |
| 153 | 45.6 | 1 | 77 | 1 | 1 | 173 | 86 | 42.75145 | 1 | 50 | 40 | 17 | 5050 |
| 154 | 45.88 | 1 | 78 | 2 | 2 | 172 | 88 | 45.02326 | 2 | 61 | 241 | 65 | 2691 |
| 155 | 45.9 | 2 | 85 | 1 | 2 | 176 | 85 | 41.05114 | 2 | 15 | 30 | 0.13 | 8096 |
| 156 | 46.38 | 1 | 87 | 1 | 1 | 165 | 85 | 43.78788 | 2 | 11 | 12 | 0.44 | 5349 |
| 157 | 46.72 | 2 | 61 | 2 | 2 | 158 | 55.5 | 19.49525 | 1 | 9 | 13 | 1.67 | 724.4 |
| 158 | 50.45 | 2 | 78 | 1 | 1 | 168 | 65 | 25.14881 | 2 | 120 | 233 | 80 | 17380 |
| 159 | 50.6 | 1 | 60 | 1 | 1 | 173 | 75 | 32.51445 | 2 | 60 | 218 | 80 | 426.7 |
| 160 | 51.3 | 1 | 74 | 1 | 1 | 172 | 70 | 28.48837 | 2 | 19 | 70 | 1.62 | 2833 |
| 161 | 53.69 | 1 | 65 | 1 | 2 | 171 | 56 | 18.33918 | 2 | 83 | 476 | 80 | 3253 |
| 162 | 55.14 | 1 | 50 | 1 | 2 | 175 | 100 | 57.14286 | 1 | 89 | 430 | 80 | 2145 |
| 163 | 55.2 | 2 | 62 | 1 | 2 | 160 | 60 | 22.5 | 2 | 43 | 112 | 23.8 | 3032 |
| 164 | 56.03 | 1 | 63 | 2 | 2 | 170 | 73.3 | 31.60524 | 2 | 17 | 25 | 0.14 | 5953 |
| 165 | 56.5 | 1 | 48 | 2 | 1 | 179 | 84 | 39.41899 | 2 | 50 | 26 | 14.29 | 4093 |
| 166 | 58.7 | 1 | 88 | 1 | 1 | 170 | 75 | 33.08824 | 2 | 55 | 73 | 2.22 | 2529 |
| 167 | 59.1 | 1 | 52 | 2 | 1 | 169 | 85 | 42.75148 | 1 | 45 | 98 | 68 | 4142 |
| 168 | 62.26 | 1 | 26 | 2 | 2 | 160 | 75 | 35.15625 | 1 | 11 | 19 | 0.45 | 1242 |
| 169 | 62.3 | 1 | 72 | 1 | 1 | 189 | 55 | 16.00529 | 2 | 52 | 76 | 7.06 | 2873 |
| 170 | 64.2 | 1 | 78 | 1 | 2 | 166 | 56 | 18.89157 | 2 | 50 | 71 | 22.9 | 31935 |
| 171 | 67.7 | 1 | 48 | 1 | 1 | 169 | 90 | 47.92899 | 2 | 18 | 16 | 20.12 | 1512 |
| 172 | 68 | 1 | 78 | 1 | 2 | 167 | 67 | 26.88024 | 1 | 49 | 36 | 59 | 4406 |
| 173 | 70.26 | 1 | 74 | 1 | 1 | 175 | 64 | 23.40571 | 2 | 26 | 88 | 37.5 | 5363 |
| 174 | 70.58 | 1 | 67 | 1 | 2 | 178 | 66 | 24.47191 | 1 | 18 | 15 | 0.03 | 412.9 |
| 175 | 72.3 | 2 | 79 | 1 | 1 | 162 | 67 | 27.70988 | 1 | 15 | 14 | 78 | 1783 |
| 176 | 73.83 | 1 | 59 | 1 | 2 | 175 | 76 | 33.00571 | 1 | 68 | 65 | 66.7 | 2496 |
| 177 | 75.9 | 1 | 71 | 2 | 2 | 165 | 62 | 23.29697 | 2 | 48 | 249 | 23.1 | 1391 |
| 178 | 76.37 | 1 | 55 | 2 | 1 | 165 | 70 | 29.69697 | 2 | 53 | 186 | 60.4 | 3015 |
| 179 | 76.53 | 2 | 74 | 1 | 1 | 183 | 90 | 44.2623 | 2 | 32 | 137 | 36.9 | 11076 |
| 180 | 79.31 | 2 | 84 | 1 | 1 | 155 | 50 | 16.12903 | 1 | 15 | 23 | 0.09 | 2500 |
| 181 | 79.9 | 1 | 79 | 1 | 1 | 165 | 72 | 31.41818 | 1 | 11 | 19 | 2.31 | 1810 |
| 182 | 83 | 2 | 64 | 2 | 1 | 157 | 45 | 12.89809 | 2 | 29 | 25 | 73 | 22925 |
| 183 | 84.38 | 2 | 84 | 2 | 2 | 150 | 40 | 10.66667 | 2 | 32 | 120 | 25.5 | 4339 |
| 184 | 85.54 | 1 | 88 | 1 | 1 | 164 | 50 | 15.2439 | 1 | 20 | 49 | 5.97 | 6243 |
| 185 | 86.3 | 1 | 76 | 2 | 2 | 163 | 98.6 | 59.64393 | 1 | 25 | 34 | 65 | 22220.9 |
| 186 | 87.72 | 1 | 71 | 1 | 2 | 170 | 68 | 27.2 | 1 | 17 | 16 | 0.46 | 1955 |
| 187 | 90.03 | 1 | 45 | 2 | 1 | 172 | 70 | 28.48837 | 1 | 18 | 15 | 0.59 | 213.4 |
| 188 | 93 | 2 | 82 | 2 | 1 | 150 | 57 | 21.66 | 2 | 20 | 40 | 35 | 14284 |

| | | | | | | | | | | | | | | |
|---|---|---|---|---|---|---|---|---|---|---|---|---|---|---|
| 189 | 93.6 | 2 | 84 | 2 | 2 | 156 | 55 | 19.39103 | 1 | 10 | 20 | 0.01 | 7846 |
| 190 | 101.6 | 1 | 75 | 1 | 1 | 180 | 64 | 22.75556 | 1 | 79 | 290 | 0.5 | 4971 |
| 191 | 101.9 | 1 | 86 | 2 | 2 | 172 | 80 | 37.2093 | 2 | 18 | 18 | 48 | 22529 |
| 192 | 102.2 | 1 | 76 | 1 | 2 | 175 | 90 | 46.28571 | 1 | 32 | 28 | 0.1 | 3464 |
| 193 | 103.4 | 1 | 58 | 2 | 2 | 169 | 81.5 | 39.30325 | 1 | 45 | 35 | 0.01 | 4007.1 |
| 194 | 125.5 | 1 | 51 | 2 | 1 | 173 | 75 | 32.51445 | 1 | 34 | 58 | 28.4 | 1826 |
| 195 | 125.5 | 1 | 66 | 1 | 1 | 163 | 71 | 30.92638 | 1 | 22 | 25 | 0.03 | 9839 |
| 196 | 166.7 | 2 | 74 | 1 | 1 | 160 | 48 | 14.4 | 2 | 67 | 61 | 59 | 35000 |
| 197 | 168.8 | 1 | 80 | 1 | 1 | 173 | 80 | 36.99422 | 1 | 147 | 352 | 44 | 33754 |
| 198 | 169.1 | 2 | 80 | 2 | 1 | 155 | 45 | 13.06452 | 2 | 31 | 100 | 72.9 | 9337 |
| 199 | 187.8 | 1 | 80 | 1 | 1 | 166 | 76 | 34.79518 | 1 | 11 | 21 | 0.02 | 7652 |
| 200 | 196 | 1 | 72 | 1 | 1 | 173 | 71 | 29.13873 | 1 | 39 | 63 | 0.02 | 11079 |
| 201 | 222 | 1 | 60 | 1 | 2 | 172 | 90 | 47.09302 | 1 | 66 | 32 | 0.11 | 70.5 |
| 202 | 323.4 | 1 | 62 | 2 | 1 | 160 | 43 | 11.55625 | 2 | 18 | 96 | 24.3 | 13228 |

| Scr | Ccr | MACE in 6 months （1=yes，2=no） | EPCI （1=yes | LDL-C |
|---|---|---|---|---|
| 53.2 | 57.4016 | 1 | 1 | 4.33 |
| 70 | 66.01467 | 1 | 2 | 4.32 |
| 61 | 81.03431 | 1 | 1 | 4.26 |
| 72 | 65.11478 | 1 | 2 | 1.64 |
| 26 | 149.7555 | 1 | 2 | 5.73 |
| 82.9 | 63.75254 | 1 | 2 | 5.25 |
| 78.1 | 82.19354 | 1 | 2 | 4.98 |
| 69 | 85.04305 | 1 | 2 | 4.98 |
| 96 | 52.72005 | 1 | 2 | 4.26 |
| 77.6 | 57.2855 | 1 | 1 | 4.98 |
| 57 | 127.1608 | 1 | 2 | 4.81 |
| 53.9 | 43.37699 | 1 | 1 | 6.5 |
| 85.8 | 54.25707 | 1 | 2 | 4.45 |
| 40 | 130.5012 | 1 | 2 | 3.64 |
| 65 | 63.78597 | 1 | 1 | 5.23 |
| 69.5 | 76.34167 | 1 | 1 | 6.75 |
| 56 | 75.5403 | 1 | 1 | 4.85 |
| 70.1 | 76.96305 | 1 | 1 | 4.89 |
| 49 | 124.0582 | 1 | 1 | 4.27 |
| 96 | 71.71964 | 1 | 2 | 4.26 |
| 51 | 123.6876 | 1 | 2 | 2.28 |
| 83.9 | 61.54725 | 1 | 2 | 5 |
| 75 | 72.76284 | 1 | 2 | 4.53 |
| 55.5 | 48.02419 | 1 | 1 | 3.45 |
| 37.3 | 150.937 | 1 | 1 | 3.84 |
| 45 | 104.1836 | 1 | 2 | 5.92 |
| 93 | 98.91684 | 1 | 2 | 2.14 |
| 55.6 | 133.2211 | 1 | 2 | 3.19 |
| 159 | 36.67482 | 1 | 2 | 4.6 |
| 78 | 88.95994 | 1 | 2 | 4.1 |
| 85.6 | 84.97475 | 1 | 2 | 2.07 |
| 119 | 46.47531 | 1 | 2 | 3.23 |
| 178.8 | 20.30653 | 1 | 2 | 5.21 |
| 93.5 | 67.77977 | 1 | 2 | 5.7 |
| 71 | 44.9895 | 1 | 1 | 2.21 |
| 47 | 165.6869 | 1 | 2 | 4.26 |

| | | | | |
|---|---|---|---|---|
| 88 | 68.87642 | 1 | 1 | 6.58 |
| 137.8 | 46.13184 | 1 | 2 | 5.56 |
| 255 | 17.70579 | 1 | 1 | 3.91 |
| 74 | 25.94991 | 1 | 1 | 1.69 |
| 43 | 125.3767 | 1 | 1 | 2.91 |
| 71 | 116.223 | 1 | 2 | 3.55 |
| 71 | 82.3892 | 1 | 2 | 6.31 |
| 87 | 77.90237 | 1 | 1 | 5.49 |
| 106 | 42.34903 | 1 | 1 | 2.25 |
| 237 | 31.05238 | 1 | 2 | 5.29 |
| 85 | 64.43262 | 1 | 2 | 5.58 |
| 64 | 119.9381 | 2 | 1 | 3.11 |
| 57.7 | 135.5972 | 2 | 2 | 3.23 |
| 56 | 121.1142 | 2 | 1 | 2.38 |
| 44 | 121.5298 | 2 | 2 | 2.85 |
| 107.1 | 46.0233 | 2 | 1 | 3.77 |
| 65.6 | 114.2734 | 2 | 1 | 3.67 |
| 74 | 74.73733 | 2 | 2 | 3.64 |
| 87 | 44.05193 | 2 | 1 | 1.44 |
| 53 | 85.75679 | 2 | 2 | 5.97 |
| 76.8 | 80.22616 | 2 | 1 | 4.03 |
| 73 | 101.3163 | 2 | 1 | 6.61 |
| 76 | 57.50547 | 2 | 1 | 4.88 |
| 84.6 | 58.44128 | 2 | 1 | 3.94 |
| 64 | 165.2659 | 2 | 1 | 3.03 |
| 72.9 | 79.77234 | 2 | 1 | 6.45 |
| 53 | 142.1322 | 2 | 2 | 5.95 |
| 72.9 | 86.09443 | 2 | 1 | 4.99 |
| 75.4 | 96.62404 | 2 | 1 | 6.86 |
| 83 | 75.34245 | 2 | 1 | 4.5 |
| 59 | 125.0466 | 2 | 1 | 2.46 |
| 72 | 51.23438 | 2 | 1 | 5.95 |
| 77.4 | 80.07809 | 2 | 1 | 4.54 |
| 66 | 133.363 | 2 | 2 | 5.85 |
| 81 | 93.95092 | 2 | 1 | 5.17 |
| 50 | 171.6381 | 2 | 1 | 1.71 |
| 84.5 | 60.82377 | 2 | 2 | 4.8 |
| 138 | 25.76716 | 2 | 1 | 2.05 |

| | | | | |
|---|---|---|---|---|
| 53.3 | 144.8185 | 2 | 1 | 5.01 |
| 69.7 | 68.40353 | 2 | 1 | 4.46 |
| 56.6 | 109.3549 | 2 | 1 | 4.63 |
| 85 | 68.66101 | 2 | 1 | 5.6 |
| 77 | 101.6099 | 2 | 2 | 4.36 |
| 57 | 125.4665 | 2 | 1 | 2.88 |
| 65.4 | 87.70552 | 2 | 2 | 6.85 |
| 70.6 | 76.79894 | 2 | 1 | 4.42 |
| 71 | 75.41582 | 2 | 1 | 6.54 |
| 75.9 | 89.29521 | 2 | 1 | 3.58 |
| 80 | 82.36553 | 2 | 1 | 4.79 |
| 72 | 135.4931 | 2 | 1 | 4.11 |
| 43 | 113.0949 | 2 | 2 | 4.49 |
| 70 | 63.6832 | 2 | 2 | 3.87 |
| 53 | 91.75624 | 2 | 2 | 4.32 |
| 122 | 45.09199 | 2 | 1 | 3.46 |
| 67.3 | 65.80214 | 2 | 1 | 3.96 |
| 67 | 86.87005 | 2 | 1 | 5.69 |
| 47 | 102.8065 | 2 | 1 | 3.74 |
| 69.9 | 104.0606 | 2 | 1 | 6.86 |
| 69.6 | 102.7922 | 2 | 1 | 4.07 |
| 89.5 | 71.38272 | 2 | 1 | 3.07 |
| 48.8 | 100.956 | 2 | 1 | 4.65 |
| 80 | 72.50917 | 2 | 1 | 5.61 |
| 88.5 | 92.38462 | 2 | 1 | 4.82 |
| 91 | 84.63419 | 2 | 2 | 5.89 |
| 75.1 | 112.3685 | 2 | 1 | 4.45 |
| 83 | 56.46302 | 2 | 2 | 6.13 |
| 47 | 116.5271 | 2 | 1 | 3.2 |
| 66 | 86.9082 | 2 | 1 | 5.32 |
| 51.8 | 113.7297 | 2 | 2 | 4.2 |
| 69 | 100.1222 | 2 | 1 | 5.28 |
| 72 | 102.2141 | 2 | 1 | 6.75 |
| 75 | 78.40261 | 2 | 1 | 3.96 |
| 63 | 138.5105 | 2 | 1 | 4.77 |
| 66 | 84.27799 | 2 | 1 | 4.77 |
| 93.05 | 71.73366 | 2 | 1 | 6.12 |
| 133 | 41.17874 | 2 | 1 | 4.95 |

| | | | | |
|---|---|---|---|---|
| 146.1 | 44.28089 | 2 | 1 | 3.04 |
| 71 | 96.23265 | 2 | 2 | 5.39 |
| 58.6 | 94.9206 | 2 | 1 | 4.45 |
| 50.7 | 151.0395 | 2 | 1 | 6.26 |
| 65 | 70.50028 | 2 | 2 | 4.46 |
| 64.8 | 66.04184 | 2 | 2 | 4.17 |
| 50.7 | 130.5561 | 2 | 1 | 4.54 |
| 61.1 | 140.6568 | 2 | 1 | 6.64 |
| 75 | 145.2323 | 2 | 1 | 6.28 |
| 71 | 167.0168 | 2 | 1 | 2.82 |
| 61 | 153.3128 | 2 | 1 | 2.38 |
| 62.9 | 122.8325 | 2 | 2 | 5.1 |
| 101 | 60.24111 | 2 | 2 | 6.48 |
| 83 | 98.31502 | 2 | 1 | 3.74 |
| 111 | 50.11124 | 2 | 1 | 5.06 |
| 64 | 137.7216 | 2 | 1 | 4.88 |
| 55.2 | 117.3727 | 2 | 1 | 4.42 |
| 66 | 109.654 | 2 | 1 | 5.59 |
| 68 | 99.32763 | 2 | 1 | 5.32 |
| 56 | 111.7709 | 2 | 1 | 4.45 |
| 65.6 | 49.80172 | 2 | 1 | 3.21 |
| 43 | 113.0949 | 2 | 1 | 3.57 |
| 123.4 | 61.66957 | 2 | 1 | 6.05 |
| 99.6 | 44.92297 | 2 | 1 | 5.23 |
| 76.8 | 54.32378 | 2 | 1 | 4.8 |
| 78 | 106.5764 | 2 | 2 | 1.73 |
| 55.5 | 48.02419 | 2 | 2 | 6.22 |
| 55 | 145.0322 | 2 | 1 | 4.76 |
| 78 | 91.68704 | 2 | 1 | 1.07 |
| 72.7 | 85.75954 | 2 | 2 | 2.96 |
| 98.6 | 54.75186 | 2 | 1 | 2.4 |
| 77.9 | 95.88495 | 2 | 1 | 4.14 |
| 57 | 106.5929 | 2 | 1 | 2.45 |
| 59 | 112.2622 | 2 | 1 | 5.1 |
| 37.9 | 123.3783 | 2 | 1 | 3.8 |
| 44 | 145.2406 | 2 | 2 | 5 |
| 59 | 128.4655 | 2 | 1 | 3.35 |
| 73 | 53.55528 | 2 | 1 | 5.33 |

| | | | | |
|---|---|---|---|---|
| 51 | 111.2469 | 2 | 1 | 4.85 |
| 44 | 84.74105 | 2 | 1 | 6.15 |
| 115.5 | 57.34608 | 2 | 1 | 2.9 |
| 103 | 64.75657 | 2 | 1 | 6.35 |
| 65 | 74.73669 | 2 | 2 | 3.5 |
| 85 | 64.79218 | 2 | 2 | 2.97 |
| 53.6 | 85.00039 | 2 | 2 | 2.87 |
| 59.5 | 70.38072 | 2 | 1 | 6.09 |
| 96 | 76.40587 | 2 | 2 | 5.74 |
| 148 | 38.16163 | 2 | 1 | 2.64 |
| 79 | 64.99335 | 2 | 1 | 4.95 |
| 83 | 132.5596 | 2 | 1 | 3.09 |
| 43 | 113.0949 | 2 | 1 | 5.74 |
| 83.1 | 83.03102 | 2 | 1 | 4.16 |
| 88.6 | 106.6302 | 2 | 2 | 5.12 |
| 391 | 12.19367 | 2 | 2 | 2.07 |
| 65 | 140.6808 | 2 | 1 | 4.53 |
| 96.6 | 108.2021 | 2 | 1 | 5.86 |
| 71 | 64.39616 | 2 | 1 | 5.02 |
| 119.1 | 35.63811 | 2 | 1 | 5.35 |
| 78 | 129.7724 | 2 | 2 | 2.78 |
| 89 | 57.05887 | 2 | 1 | 4.3 |
| 70 | 73.76877 | 2 | 1 | 5.32 |
| 57 | 103.3329 | 2 | 1 | 2.63 |
| 285 | 14.90134 | 2 | 1 | 6.78 |
| 79 | 95.26168 | 2 | 2 | 2.08 |
| 70 | 74.71184 | 2 | 1 | 5.09 |
| 75.4 | 96.47001 | 2 | 1 | 3.52 |
| 68 | 90.77017 | 2 | 1 | 5.98 |
| 65.7 | 44.28517 | 2 | 2 | 5.85 |
| 87 | 61.71486 | 2 | 1 | 5.2 |
| 84.1 | 42.25671 | 2 | 1 | 4.85 |
| 53 | 43.91752 | 2 | 2 | 4.59 |
| 53 | 59.9714 | 2 | 1 | 4.88 |
| 98.6 | 78.23961 | 2 | 1 | 3.29 |
| 88 | 65.18115 | 2 | 1 | 3.94 |
| 71.3 | 114.0194 | 2 | 1 | 1.41 |
| 79.2 | 43.37538 | 2 | 1 | 2.85 |

| | | | | |
|---|---|---|---|---|
| 83 | 38.56011 | 2 | 2 | 2.59 |
| 115 | 44.22239 | 2 | 2 | 3.12 |
| 194 | 27.22254 | 2 | 2 | 5.88 |
| 70.1 | 100.4503 | 2 | 2 | 5.09 |
| 61 | 133.9332 | 2 | 2 | 2.57 |
| 55.5 | 147.0297 | 2 | 1 | 3.98 |
| 170.2 | 37.73785 | 2 | 1 | 3.82 |
| 262 | 12.56462 | 2 | 1 | 4.16 |
| 144 | 40.7498 | 2 | 1 | 4.03 |
| 70.6 | 39.73971 | 2 | 1 | 4.18 |
| 96.5 | 57.76759 | 2 | 1 | 6.14 |
| 93 | 63.46452 | 2 | 1 | 2.61 |
| 50.8 | 173.2669 | 2 | 2 | 2.72 |
| 82.5 | 49.69993 | 2 | 1 | 4.66 |
